# A novel host restriction factor MRPS6 mediates the inhibition of PDCoV infection in HIEC-6 cells

**DOI:** 10.3389/fimmu.2024.1381026

**Published:** 2024-06-11

**Authors:** Yuhang Jiang, Guoqing Zhang, Letian Li, Jing Chen, Pengfei Hao, Zihan Gao, Jiayi Hao, Zhiqiang Xu, Maopeng Wang, Chang Li, Ningyi Jin

**Affiliations:** ^1^ College of Veterinary Medicine, Northwest A&F University, Yangling, China; ^2^ Research Unit of Key Technologies for Prevention and Control of Virus Zoonoses, Chinese Academy of Medical Sciences, Changchun Institute of Veterinary Medicine, Chinese Academy of Agricultural Sciences, Changchun, China; ^3^ Wenzhou Key Laboratory for Virology and Immunology, Institute of Virology, Wenzhou University, Wenzhou, China

**Keywords:** PDCoV, proteomics, MRPS6, host restriction factor, IFN-β

## Abstract

**Introduction:**

Porcine deltacoronavirus (PDCoV) is a zoonotic pathogen with a global distribution, capable of infecting both pigs and humans. To mitigate the risk of cross-species transmission and potential outbreaks, it is crucial to characterize novel antiviral genes, particularly those from human hosts.

**Methods:**

This research used HIEC-6 to investigate PDCoV infection. HIEC-6 cells were infected with PDCoV. Samples were collected 48 h postinfection for proteomic analysis.

**Results:**

We discovered differential expression of MRPS6 gene at 48 h postinfection with PDCoV in HIEC-6 cells. The gene expression initially increased but then decreased. To further explore the role of MRPS6 in PDCoV infection, we conducted experiments involving the overexpression and knockdown of this gene in HIEC-6 and Caco2 cells, respectively. Our findings revealed that overexpression of MRPS6 significantly inhibited PDCoV infection in HIEC-6 cells, while knockdown of MRPS6 in Caco2 cells led to a significant increase of virus titer. Furthermore, we investigated the correlation between PDCoV infection and the expression of MRPS6. Subsequent investigations demonstrated that MRPS6 exerted an augmentative effect on the production of IFN-β through interferon pathway activation, consequently impeding the progression of PDCoV infection in cellular systems. In conclusion, this study utilized proteomic analysis to investigate the differential protein expression in PDCoV-infected HIEC-6 cells, providing evidence for the first time that the MRPS6 gene plays a restrictive role in PDCoV virus infection.

**Discussion:**

Our findings initially provide the validation of MRPS6 as an upstream component of IFN-β pathway, in the promotion of IRF3, IRF7, STAT1, STAT2 and IFN-β production of HIEC-6 via dual-activation from interferon pathway.

## 1 Introduction

Coronaviruses (CoV) belong to a group of enveloped RNA viruses classified into four genera: alpha, beta, gamma, and delta. These viruses can induce respiratory and gastrointestinal diseases in both human and animal populations, with symptoms ranging in severity (1). Severe Acute Respiratory Syndrome Coronavirus (SARS-CoV), Middle East Respiratory Syndrome Coronavirus (MERS-CoV), and Severe Acute Respiratory Syndrome Coronavirus 2 (SARS-CoV-2) gave rise to significant epidemics with notable levels of morbidity and mortality in the years 2003, 2012, and 2019, respectively (2). Over 675 instances of animal outbreaks have been documented, leading to a total of over 2,000 animal infections, encompassing both domestic and wild species. (3). Civets as a direct source of SARS-CoV-2 (4). Non-primate model animals, including cats and dogs (5) ferrets (6) monkeys (7) baboons (8) etc. This suggests that SARS-CoV-2 can be transmitted between humans and animals. MERS is an exceedingly fatal respiratory ailment induced by a distinct *betacoronavirus* member known as MERS-CoV (9). The MERS-CoV similar to several other coronaviruses, originates in bats (10). Human transmission of MERS-CoV through the intermediary role of infected dromedary camels (11). The inaugural instance of human infection with the MERS-CoV was documented in the year 2012 (12). Coronaviruses possessing zoonotic potential entail consequential biosafety hazards, thereby presenting noteworthy disease burdens and economic ramifications for society.

PDCoV is a coronavirus that was initially discovered in Hong Kong, China in 2012 (13). Since then, outbreaks of PDCoV have been reported in the United States (14) and various countries in Asia (15–17). PDCoV can infect pigs of all ages, but piglets are more susceptible to the virus (18). The primary sites of infection are the distal jejunum and ileum, leading to symptoms such as watery diarrhea, vomiting, dehydration, and weight loss (19, 20). Their clinical manifestations are similar to those caused by porcine epidemic diarrhea virus (PEDV), transmissible gastroenteritis virus (TGEV), and co-infections (21). This virus poses a significant threat to the livestock industry, particularly due to its high piglet mortality rates. PDCoV exhibits a broad spectrum of infectivity across multiple species, including pigs, poultry, mice, leopard cats, pigeons, and even humans, indicating a potential risk to public health (21–26).

The entry of PDCoV into host cells is mediated by aminopeptidase N, which plays a crucial role in facilitating viral entry through the endocytosis pathway. Endocytic viral entry is a crucial determinant of the efficient replication of PDCoV (27). Its infection results in a decrease in peroxisome abundance, which serves as the site for MAVS activation, leading to the induction of IRF1 and subsequent production of type III interferon (IFN). Furthermore, PDCoV actively suppresses the type III IFN response as a strategy to evade host antiviral immunity (28). Ergosterol peroxide (EP) demonstrates inhibitory effects on PDCoV infection and regulates the immune responses of the host by suppressing the activation of NF-κB and p38/MAPK signaling pathways. HIEC cells, derived from the fetal immature small intestine, are susceptible to PDCoV infection (29), indicating its potential to spread disease among individuals (30). However, the precise mechanism underlying its cross-species infection remains unclear. To address this knowledge gap, the present study employed HIEC-6 as a cell model and conducted a comprehensive proteomic analysis. The aim was to elucidate the novel molecular mechanisms associated with PDCoV infection at the protein level and identify potential antiviral agents with efficacy against this pathogen.

Differential proteomics has emerged as a valuable tool for studying virus-host interactions and the modulation of cellular processes during infection (31). By investigating virus-host protein interactions, proteomics provides insights into the molecular mechanisms of viral replication and pathogenesis (32). While numerous inhibitory host proteins have been identified for PDCoV infection in pigs, there is a lack of specific studies investigating the relationship with human proteins or innate immune molecules. Thus, this experiment aims to provide a preliminary investigation into the association between the differential expression gene (DEG) MRPS6 and PDCoV infection in humans.

## 2 Materials and methods

### 2.1 Cells and viruses

HIEC-6, MA104, HEK293, HEK293T, DF1, MDCK, BHK21, ST, LLC-PK1, IPEC-J2, and Caco2 were kept in our laboratory(The cell lines present in this study were obtained from ATCC). All cells were cultured using Dulbecco’s Modified Eagle’s Medium (DMEM; Gibco) containing 10% fetal bovine serum (Gibco) and 1% penicillin-streptomycin solution (Hyclone). PDCoV (Genbank No. OK546242) was a kind gift from Prof. Bin Li (33).

### 2.2 Antibodies and reagents

HRP-conjugated goat anti-rabbit IgG (H+L) (CAT#A0208), Cy3-labeled goat anti-rabbit immunoglobulin (H+L) (Cat #A0516) and FITC-labeled goat anti-mouse immunoglobulin (H+L) (CAT#A0562) were purchased from Beyotime Biotech Inc. Lipo™ RNAiMAX and LIpo™ 3000 were purchased from Invitrogen. SYBR-Green (Promega) and Poly-IC (Merck, USA) were used in the experiments. Anti-PDCoV N rabbit polyclonal antibody was prepared in our laboratory. Specific antibodies for MRPS6 and GAPDH were separately purchased from ORIGENE and Genetex. Polyclonal antibody for IRF-3 was purchased from Sanying (Proteintech). Antibody for STAT1, STAT2, IRF7 and IFN-Beta was purchased from Cell Signaling Technology.

pCAGGS-MRPS6 was constructed in our laboratory. Briefly, MRPS6 was obtained by amplification using primers and pCAGGS-MRPS6 was obtained by subcloning *EcoR I* and Not *I* into pCAGGS (Inovogen Technologies). Recombinant plasmids were validated by PCR and sequencing.

### 2.3 Virus replication kinetics

LLC-PK1 cells were inoculated with PDCoV for virus replication. The virus was subsequently diluted with DMEM containing 10 μg/mL trypsin. After incubation at 37°C for 2 hours, the medium of host cell LLC-PK1 was changed to remove unattached virus until 50% cell were induced to produce cytopathic effect (CPE). The cells were then subjected to one cycle of freeze-thawing, and the supernatant containing the virus was collected by centrifugation at 12,000 rpm for 1 min.

### 2.4 One-step growth curve

HIEC-6 cells were seeded into 12-well plates at a concentration of 2 × 10^5^ cells per well, incubated overnight to reach 70%–80% confluency, and infected with the PDCoV virus at a MOI of 1. The cell-virus admixture was harvested at time intervals of 6, 12, 24, 36, 48 and 60 hours postinfection. Each collected sample underwent a single freeze-thaw cycle, followed by centrifugation at 12,000 rpm for 1 min to precipitate cellular debris, thereby allowing for the isolation of the viral supernatant.

### 2.5 Virus titer assay

We have determined the virus titer as previous description (34). Briefly, HIEC-6 or Caco2 cells were seeded in 96-well plates at a density of 1×10^5^ cells per well. The PDCoV samples were then diluted serially at a 10-fold dilution with 100 µL per well, and each sample was repeated three times. The TCID_50_ (median tissue culture infectious dose) was subsequently calculated using the Reed-Muench method.

### 2.6 Plasmids, siRNA, poly-IC transfection

The coding sequence of human MRPS6 (NM_032476.4) was amplified from Caco2 cells and subsequently inserted into the pCAGGS vector. HIEC-6 cells were cultured until reaching 80% confluency and transfected with the pCAGGS-MRPS6 plasmid or poly-IC (60 mM per well) using LIpo™ 3000 Transfection Reagent. MRPS6 siRNA(stB0013147A) purchased from RiboBio was transfected into Caco2 with 80–90% confluency with RNAiMAX transfection reagent for preparation.

### 2.7 Mass spectrometry analysis, data processing of proteomics

The protein samples were subjected to extraction and subsequently underwent quality control. Those samples that passed the quality control were then separated and subjected to liquid quality control in DDA mode. The obtained data from DDA scanning mode were searched against the Human Protein Data Bank using Spectronaut-Pulsar (version 14.0, Biognosys), a library search software. Once the search was completed, Spectronaut software (version 14.0, Biognosys) was utilized to directly generate spectral libraries. Additionally, interscan software was employed to annotate the GO functions, which included Pfam, PRINTS, ProDom, SMART, ProSite, and PANTHER databases. Functional protein family, and pathway analysis of identified proteins by KEGG (35). Volcano plot analysis, clustered heatmap analysis, and pathway enrichment analysis for DEGs (DEGs) were conducted. The Gene Ontology (GO) and Kyoto Encyclopedia of Genes and Genomes (KEGG) databases were utilized for pathway enrichment analysis. Additionally, potential protein-protein interactions (PPI) were predicted using the STRING DB software (http://STRING.embl.de/) (36).

### 2.8 Western blot

Cells were collected and lysed, and protein concentrations were determined by a bicinchoninic acid (BCA) protein assay kit (Beyotime, Cat#P0010). The proteins were mixed with loading buffer and denatured by boiling. Then, equal amounts (30 μg) were electrophoresed and transferred onto a nitrocellulose filter membrane, and then incubated overnight at 4°C with primary antibodies. After further incubation with HRP-conjugated secondary antibodies, the membranes were detected by the Amersham Imaging 600 system, with Pierce ECL Western blotting substrate (Thermo Fisher Scientific, Cat#32106) (37).

### 2.9 RT-qPCR

Total cellular RNA was extracted and quantified following the manufacturer's instructions (Sangon Biotech, Cat# B511311). Total RNA was employed as a template and subjected to reverse transcription using M-MLV reverse transcriptase (Promega) to generate complementary DNA (cDNA). The cDNA was subsequently subjected to a real-time quantitative polymerase chain reaction (RT-qPCR) using SYBR Green Master Mix mixed with primers from (**Table 1**) on a BIO-RAD CFX96 RT-qPCR system (BIO-RAD). The genes were then quantified using quantitative real-time PCR and analyzed using the 2-∆∆CT method (38).

**Table 1:** Primers for RT-qPCR in this study.

Name	Sequence
*PDCoV*-N-F	CTATGAGCCACCCACCAA
*PDCoV*-N-R	TCCCACTCCCAATCCTGT
*MRPS6*-F	ACGTACGATAGAGGCCCTGA
*MRPS6*-R	CCACCAAGAAATACCCGCCT
*IFN-β*F	TCTCCTGTTGTGCTTCTCCAC
*IFN-β*R	GCCTCCCATTCAATTGCCAC
*IRF-3*F	AGAGGCTCGTGATGGTCAAG
*IRF-3*R	AGGTCCACAGTATTCTCCAGG
*GAPDH*-F	CTACATGGTTTACATGTTCC
*GAPDH*-R	GGATCTCGCTCCTGGAAGAT

### 2.10 Immunofluorescence assay

HIEC-6 or Caco2 cell lines were seeded into 12-well plates at a density of 5 × 10^5^ cells per well. The target plasmids were transfected and cultured for 36h. Subsequently, the cells were infected with the PDCoV virus at a MOI of 1 and cultured for an additional 24 hours. Following this, the cells were fixed with 4% paraformaldehyde for 30 minutes and washed thrice with PBS. The cells were then permeabilized with 0.1% Triton X-100 and blocked with 5% skimmed milk for 1 hour. Subsequently, the cells were incubated with FITC-coupled PDCoV N protein-specific primary antibody for 2 hours at 37°C. After washing with PBS, fluorescence photography was conducted.

### 2.11 Flow cytometry

Samples were collected from different host cells at 24 hours postinfection with 1 MOI PDCoV. The cells were enzymatically digested using trypsin and subsequently resuspended. The cells were then permeabilized with 0.1% Triton X-100 and blocked with 5% skim milk for 1 hour. Subsequently, the cells were incubated with FITC-coupled PDCoV N protein-specific primary antibody for 2 hours at 37°C. After washing with PBS, fluorescence photography was conducted and analyzed by CytoFLEX flow cytometry.

### 2.12 Data analysis statistical analyses

Statistical significance between groups was determined using GraphPad Prism, version 9.3.1 Data were presented as means ± standard errors of the means (SEM) in all experiments and analyzed using a one-way ANOVA and T-test, and a P value of < 0.05 was considered to be statistically significant.

## 3 Result

### 3.1 Infection spectrum of PDCoV

**Figure 1:**
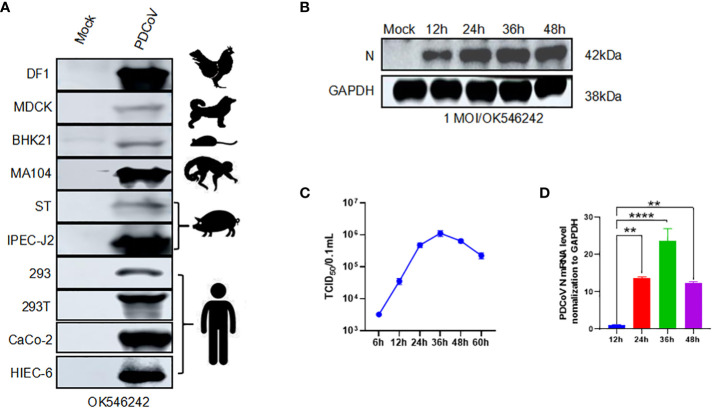
PDCoV infection modeling. We change it according to your suggestions: **Figure 1** PDCoV infection modeling. **(A)** Western blot assay of PDCoV (OK546242) infected different kinds of cells with PDCoV N as the primary antibody. **(B)** Changes in PDCoV N protein at 12h, 24h, 36h, and 48h in PDCoV-infected HIEC-6 cells. **(C)** One-step growth curve of PDCoV in HIEC-6 cells, **(D)** Relative quantification analysis of PDCoV N in HIEC-6 cells infected with PDCoV, Statistical significance is determined by one-way ANOVA ( **P<0.01; ****P<0.0001).

We evaluated the infection range of PDCoV by inoculating them into ten different cell lines (HIEC-6, MA104, HEK293, HEK293T, DF1, MDCK, BHK21, ST, IPEC-J2, and Caco2). The PDCoV N was detected using a western blot to prove the existence and infection of the virus. The specific 42kDa bands were shown in all the cells. However, we discovered a higher expression from the result of avian-derived DF1, African green monkey embryonic kidney cells derived MA104, porcine-derived IPEC-J2, and human-derived Caco2 or HIEC-6 (**Figure 1A**). Subsequently, we opted for the utilization of HIEC-6 cells, which exhibited a greater level of infection, for subsequent experiments. We conducted an evaluation of the expression of PDCoV N protein at various time points (12h, 24h, 36h, and 48h). Immunoblot analysis revealed the presence of the target strip measuring 42kDa at all time intervals, with the maximum intensity observed at 24h. Subsequently, no significant changes in protein expression were observed (**Figure 1B**). The viral titer was assessed, with the highest level observed at 36h, reaching 10^5.5^ TCID_50_/0.1ml (**Figure 1C**). To quantify the expression levels of PDCoV N, RT-qPCR was performed with GAPDH as the internal reference. The mRNA expression level PDCoV N expression level showed a significant difference at 48h (**Figure 1D**). In conclusion, PDCoV can infect HIEC-6 cells and replicate within HIEC-6.

### 3.2 PDCoV infection with HIEC-6 proteomics

**Figure 2:**
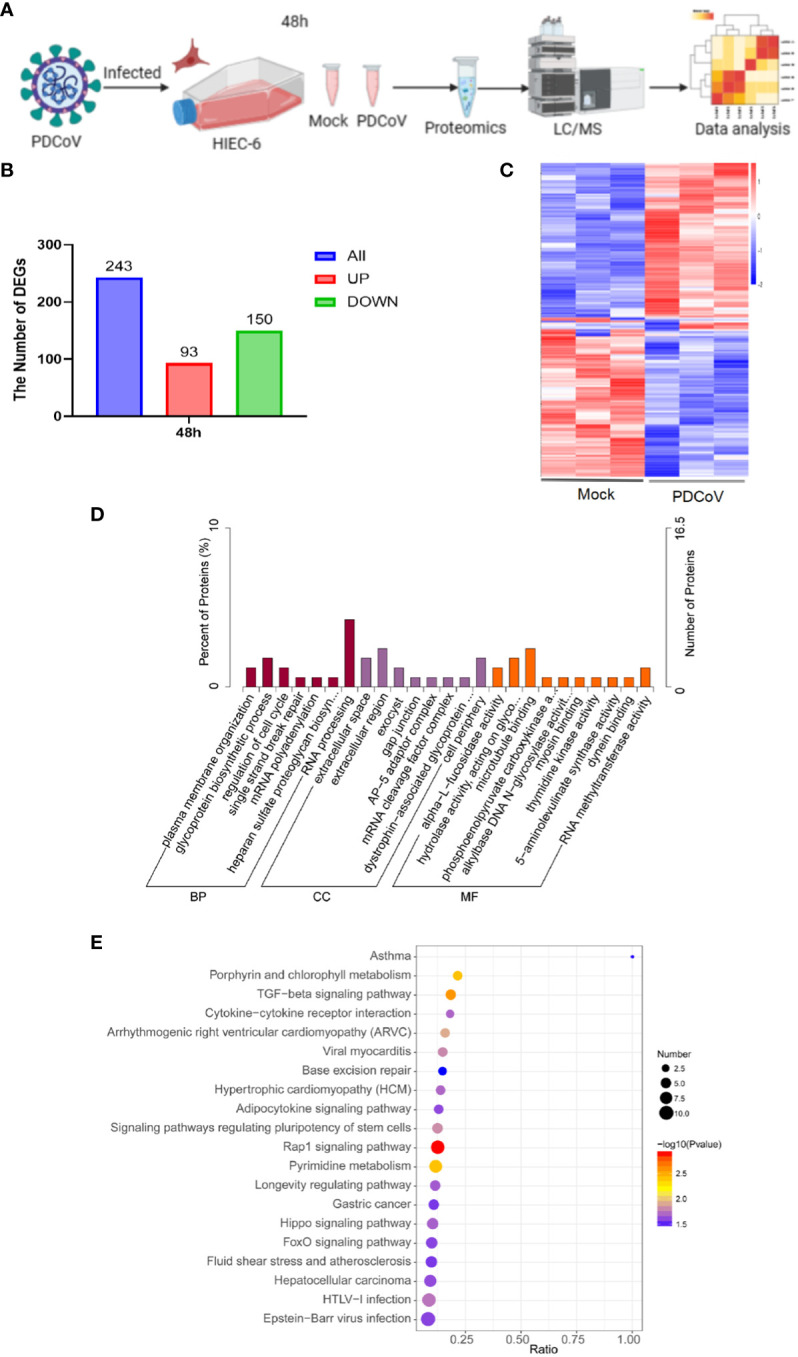
Proteomics analysis of all DEGs. **(A)** Flow of PDCoV-infected HIEC-6 cell proteomics experiments. **(B)** Number of DEGs at 48h. **(C)** Enrichment of DEGs heatmap at 48h. **(D)** GO annotation of differentially expressed genes or DEGs at 48h. **(E)** KEGG annotation of differentially expressed DEGs at 48h.

The objective of this study was to examine the impact of PDCoV infection on HIEC-6 cells after 48 hours by evaluating alterations in gene expression induced by the virus. Uninfected cells were utilized as a control group (referred to as “Mock”). The 4D-DIA technique was employed to analyze the samples (**Figure 2A**). A total of 243 DEGs were identified at 48h, with 93 DEGs being upregulated and 150 DEGs being downregulated (**Figures 2B, C** and **Supplementary Tables 1, 2**). Gene ontology (GO) enrichment analysis demonstrated that the enriched biological processes were associated with mRNA polyadenylation, RNA processing, RNA methyltransferase activity, and other related processes (**Figure 2D**). Furthermore, KEGG pathway analysis revealed enrichment of pathways linked to the Rap1 signaling pathway, cytokine-cytokine receptor interaction, and cancer (**Figure 2E**).

### 3.3 MRPS6 changes in HIEC-6 cells infected with PDCoV

**Figure 3:**
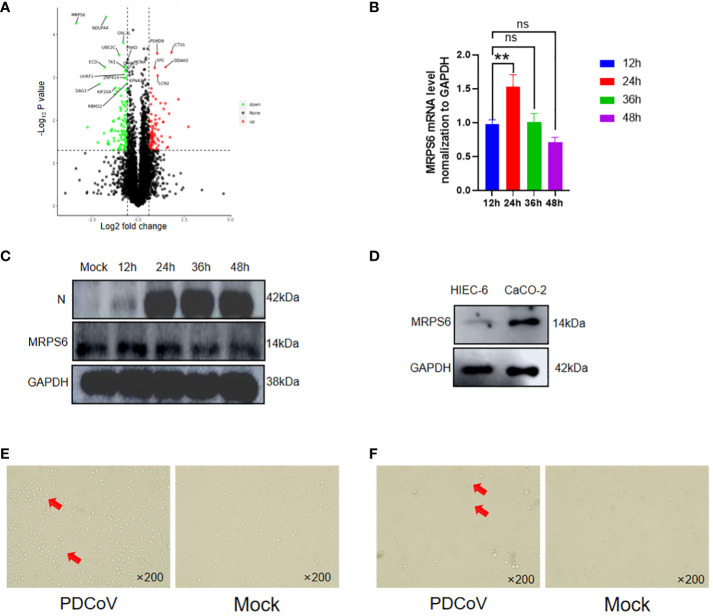
Differential Expression of MRPS6 in PDCoV-infected HIEC-6 cells. **(A)** Volcano map screening for DEGs of MRPS6. **(B)** mRNA changes of MRPS6 in HIEC-6 cells infected with PDCoV. **(C)** Changes of MRPS6 in HIEC-6 cells infected with PDCoV by western blot. **(D)** MRPS6 gene expression in HIEC-6 and Caco2 by western blot. **(E)** HIEC-6 cell lesion caused by PDCoV infection. **(F)** Caco2 cell lesions caused by PDCoV infection, Red arrow positions are cytopathic. Statistical significance is determined by one-way ANOVA (**P<0.01; n.s., not significant).

Significant differential expression of the MRPS6 gene was observed at 48 hours in PDCoV-infected HIEC-6 cells compared to uninfected cells, as determined by statistical analysis (P < 0.05) and a log2 fold change greater than 1.5. This finding was obtained through a comparative analysis of the volcanic plots, where the top 20 differentially expressed genes were identified and presented in **Figure 3A**. PDCoV-infected HIEC-6 cells were collected at different time points (12, 24, 36, and 48 hours) to investigate the expression levels of MRPS6 using RT-qPCR (**Figure 3B**). Western blot analysis (**Figure 3C**) confirmed the initial increase and subsequent decrease in MRPS6 expression from 24 to 48 hours. The highest expression of MRPS6 was observed at 24 hours of PDCoV infection. To compare MRPS6 expression between HIEC-6 and Caco2 cells, protein validation was performed, revealing significantly higher levels of MRPS6 in Caco2 cells compared to HIEC-6 cells (**Figure 3D**). Morphological analysis of PDCoV-infected HIEC-6 and Caco2 cells at 48h showed floating cells and vacuolated lesions, respectively (**Figures 3E, F**). At 48 hours post-infection, HIEC-6 cells infected with PDCoV exhibited a floating phenotype, while Caco2 cells displayed vacuolated lesions. These observations lay the groundwork for our subsequent investigations, which aim to elucidate the impact of PDCoV infection on the expression of MRPS6, evaluate the effects of MRPS6 overexpression in the HIEC-6 cell line, conduct knockdown experiments in the Caco2 cell line, and determine the TCID_50_ based on the observed cellular lesions.

### 3.4 Overexpression of MRPS6 Inhibits PDCoV proliferation

**Figure 4:**
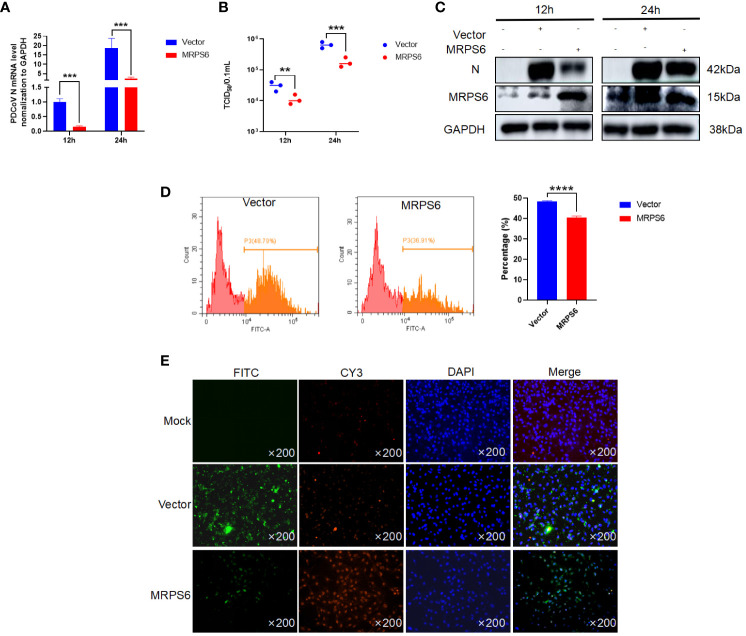
Overexpression of MRPS6 in HIEC-6 cells mediates the resistance of PDCoV infection. **(A)** Viral titers and copies are separately detected with RT-qPCR at 12 h and 24 h. **(B)** Viral titers and copies are separately detected with TCID_50_. **(C)** Immunoblotting analysis of PDCoV N indicates the infection level with or without MRPS6 overexpression at 12h and 24h. **(D–E)** Flow cytometric and immunofluorescence assay were utilized to evaluate the infection at 24 h with PDCoV N antibody as the primary antibody and Cy3-labeled goat anti-rabbit immunoglobulin (Cy3). Statistical significance is determined by one-way ANOVA (**P<0.01; ***P<0.001; ****P<0.0001).

HIEC-6 cells were utilized to investigate the overexpression of MRPS6 using the pCAGGS vector. After 36 hours of overexpression, the cells were infected with a virus at a MOI of 1. To assess the functional impact of MRPS6 overexpression, multiple experimental techniques were utilized, including Western blot analysis, RT-qPCR, TCID_50_ assay, flow cytometry, and immunofluorescence. The primary antibody employed in this study was PDCoV N, which specifically detected a band at 42kDa. The results obtained from the Western blot analysis demonstrated a significant decrease in the expression of the target band, as well as the N protein, following the overexpression of MRPS6 (**Figure 4A**). Additionally, the TCID_50_ assay revealed a reduction in viral titer in the supernatant at both 12h and 24h post-infection (**Figure 4B**). mRNA detection exhibited a notable disparity in N protein expression at both time points (**Figure 4C**). Flow cytometry analysis, utilizing FITC-coupled anti-PDCoV N antibody, exhibited a lower proportion of positive cells in HIEC-6 cells overexpressing MRPS6 in comparison to the plasmid-transfected group (**Figure 4D**). Finally, immunofluorescence results, utilizing PDCoV N as the primary antibody, indicated a significantly diminished specific fluorescence of N protein in cells overexpressing MRPS6 compared to non-transfected cells (**Figure 4E**). These findings suggest that the overexpression of MRPS6 can effectively reduce PDCoV virus infection.

### 3.5 Knockdown of MRPS6 promotes PDCoV proliferation

**Figure 5:**
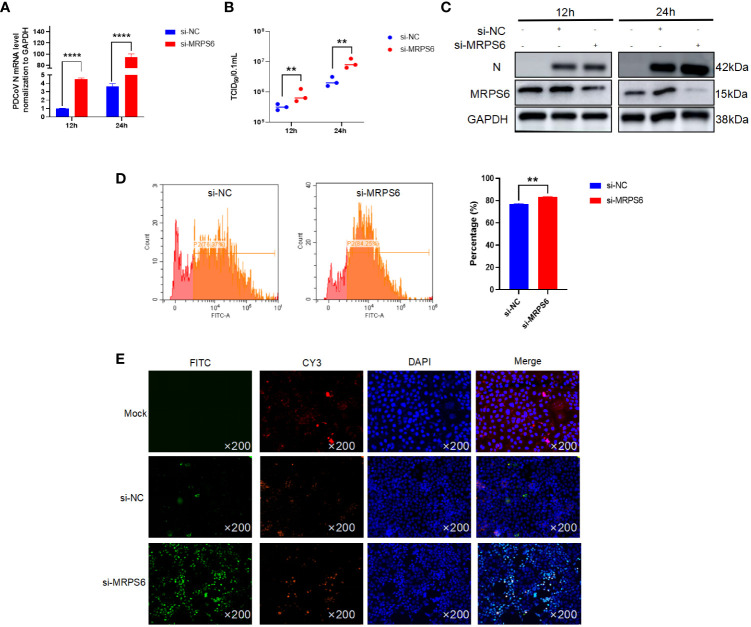
Knockdown of MRPS6 in Caco2 cells mediates the resistance of PDCoV infection. **(A)** Viral titers and copies are separately detected with RT-qPCR at 12 h and 24 h. **(B)** Viral titers and copies are separately detected with TCID_50_. **(C)** Immunoblotting analysis of PDCoV N indicates the infection level with or without Knockdown of MRPS6 at 12h and 24h. **(D–E)** Flow cytometric and immunofluorescence assay were utilized to evaluate the infection at 24h with PDCoV N antibody as the primary antibody and Cy3-labeled goat anti-rabbit immunoglobulin (Cy3). Statistical significance is determined by one-way ANOVA (**P<0.01; ****P<0.0001).

Subsequently To elucidate the functional role of MRPS6, a variety of experimental techniques were employed, including Western blot analysis, RT-qPCR, TCID_50_, flow cytometry, and immunofluorescence. The primary antibody utilized in this investigation specifically targeted the PDCoV N protein, enabling the detection of a band at a molecular weight of 42 kDa. The results obtained from the Western blot analysis revealed an upregulation in the expression of the PDCoV N protein following MRPS6 inhibition (**Figure 5A**). Additionally, the TCID_50_ assay demonstrated an increase in the viral titer in the supernatant at both 12h and 24h post-infection (**Figure 5B**). RT-qPCR analysis also exhibited a significant disparity in the expression of the N protein at both time points (**Figure 5C**). Flow cytometry analysis, utilizing a FITC-conjugated anti-PDCoV N antibody, displayed a higher percentage of positive cells in Caco2 cells with MRPS6 inhibition compared to the siRNA-NC group (**Figure 5D**). Finally, the immunofluorescence results, utilizing the PDCoV N protein as the primary antibody, revealed significantly higher specific fluorescence of N proteins in MRPS6-inhibited cells as opposed to untransfected cells (**Figure 5E**). These findings strongly indicate that MRPS6 inhibition effectively enhances PDCoV infection.

### 3.6 PDCoV replication and assembly correlates with MRPS6 expression

**Figure 6:**
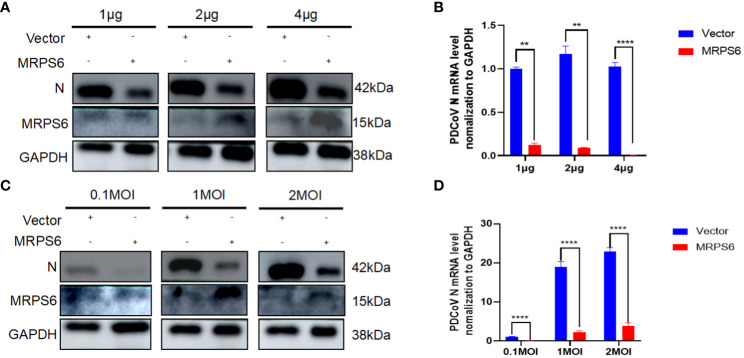
MRPS6 plasmid overexpression in HIEC-6 cells at 1 μg, 2 μg, and 4 μg following PDCoV infection using 1 MOI, MRPS6 plasmid overexpression in HIEC-6 cells at 2 μg following PDCoV viral infection using 0.1 MOI, 1 MOI, and 2MOI. **(A)** PDCoV infection using 1 MOI following MRPS6 plasmid overexpression at 1 μg, 2 μg and 4 μg, 24h immunoblotting assay of PDCoV N antibody as primary antibody **(B)** RT-qPCR assay of MRPS6 plasmid overexpression of 1 μg, 2 μg and 4 μg followed by PDCoV infection at 1 MOI, **(C)** PDCoV viral infection using MRPS6 plasmid overexpression of 2 μg in HIEC-6 cells with PDCoV N antibody at 0.1 MOI, 1 MOI and 2MOI, 24h immunoblotting assay of MRPS6 plasmid overexpression of 2 μg as primary antibody. PDCoV N antibody was used as a primary antibody in a 24-hour immunoblotting assay, **(D)** RT-qPCR assay of MRPS6 plasmid overexpressed in HIEC-6 cells for 2 μg and then infected with PDCoV virus using 0.1 MOI, 1 MOI, and 2MOI. Statistical significance is determined by one-way ANOVA (**P<0.01; ****P<0.0001).

Investigations were conducted to assess the inhibitory effects of various concentrations of MRPS6 on PDCoV infection in HIEC-6 cells. The cells were transfected with either 1 μg, 2 μg, or 4 μg of MRPS6 and subsequently infected with PDCoV. The effects were determined using Western blot and RT-qPCR techniques. Our findings demonstrated that the overexpression of 4 μg of MRPS6 significantly inhibited PDCoV viruses to a greater extent compared to the overexpression of 1 μg and 2 μg of MRPS6 (**Figures 6A, B**). The inhibitory efficacy of MRPS6 on Porcine Deltacoronavirus (PDCoV) exhibited a dose-dependent relationship, with higher concentrations demonstrating a significantly greater inhibitory effect compared to lower concentrations. To investigate the potential impact of MRPS6 on the infection of PDCoV at different MOIs, HIEC-6 cells were transfected with 4 μg of MRPS6 and subsequently infected with PDCoV at MOIs of 0.1, 1, and 2. The infection was evaluated using Western blot and RT-qPCR techniques. The results demonstrated that the overexpression of 4 μg of MRPS6 had a significantly stronger inhibitory effect on PDCoV at an MOI of 0.1 compared to an MOI of 1 and 2 (**Figures 6C, D**). These findings suggest that MRPS6 exerts a more potent inhibitory effect on PDCoV at lower MOI values.

### 3.7 Predication and verification of proteins interaction with MRPS6

**Figure 7:**
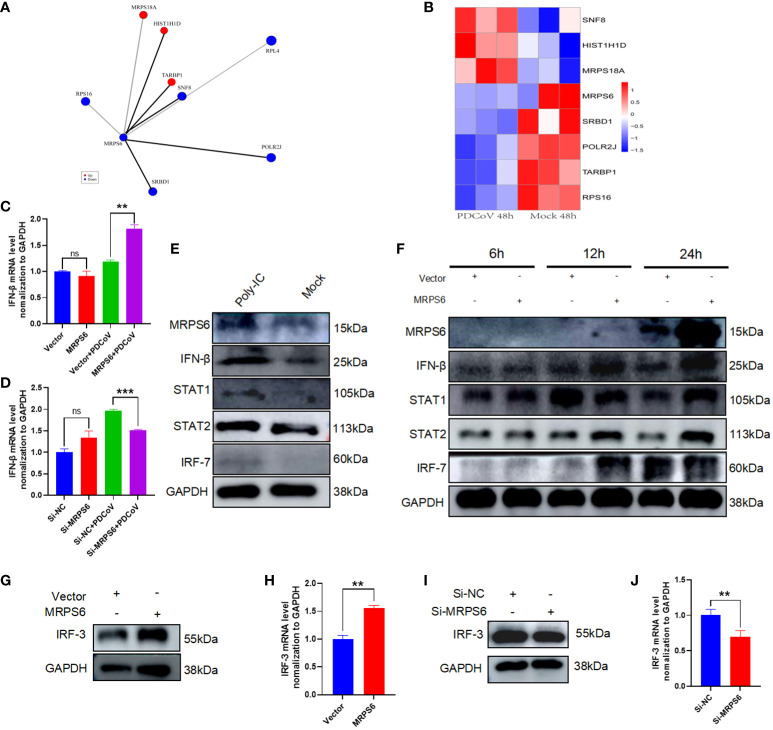
Analysis of MRPS6 interactions and speculation on MRPS6 antiviral pathway. **(A)** Proteins with significant Interaction with MRPS6. **(B)** Heatmap analysis of the expression of MRPS6-interacting proteins. **(C)** MRPS6 was overexpressed in HIEC-6 cells and infected with 1 MOI of PDCoV for 6h, and IFN-β mRNA levels were detected by RT-qPCR. **(D)** MRPS6 was knocked down in Caco2 cells and infected with 1 MOI of PDCoV for 6h, and IFN-β mRNA levels were detected by RT-qPCR. **(E)** Poly-IC stimulates activation of IFN pathway and STAT pathway in CaCo2 cells. **(F)** Overexpression of MRPS6 promotes activation of the IFN-β pathway with the JAK-STAT pathway at different times. **(G–H)** Western blot and RT-qPCR analysis showed the protein and mRNA levels of IRF-3 after 24h of transfection of the MRPS6 overexpression in HIEC-6 cells. **(I–J)** Western blot and qRT-PCR analysis revealed the protein and mRNA levels of IRF-3 after 24h of siRNA transfection in Caco2 cells. Statistical significance is determined by one-way ANOVA and T-test (**P<0.01; ***P<0.001; n.s., not significant).

Identification of MRPS6-interacting proteins was conducted using proteomic data from HIEC-6 cells infected with PDCoV. A total of eight genes were found (**Figure 7A**), with SNF8, HIST1H1D, and MRPS18A showing up-regulation, while RPS16, POLR2J, TARBP1, and RPL4 showed down-regulation (**Figure 7B**). We examined whether MRPS6 exerts regulatory effects on IFN-β in HIEC-6 cells. Following MRPS6 overexpression and PDCoV inoculation, RT-qPCR analysis revealed that MRPS6 overexpression promoted IFN-β transcription (**Figure 7C**). Subsequently, we conducted knockdown experiments on MRPS6 in Caco2 cells, which were then infected with 1MOI PDCoV. The mRNA level of IFN-β was evaluated using RT-qPCR 6 hours later. The results demonstrated a significant reduction in the mRNA level of IFN-β upon MRPS6 knockdown (**Figure 7D**). Our findings also prove that MRPS6 in the HIEC-6 cell could be induced by poly-IC, and other main components of IFN-β pathway, such as STAT1, STAT2, IRF7 and even IFN-β itself, was upregulated due to the activation role (**Figure 7E**). Further data revealed maximal expression level of MRPS6 was at 24 hours post-transfection. The expression of IFN-β, STAT1, STAT2, and IRF7 was strongly correlated with MRPS6 overexpression (**Figure 7F**). It indicates that MRPS6 can activate various downstream components from IFN-β pathway. Considering that IRF-3 is the primary transcription factor for IFN-β [38], we investigated the impact of MRPS6 on IRF-3 in HIEC-6 cell. Overexpression of MRPS6 in HIEC-6 cells resulted in increased protein and mRNA levels of IRF-3 (**Figures 7G, H**). Furthermore, we performed knockdown and overexpression experiments on MRPS6 in Caco2 cells and assessed the expression levels of IRF-3 using WB and RT-qPCR. The findings revealed a significant reduction in both the protein and mRNA levels of IRF-3 upon MRPS6 knockdown in Caco2 cells (**Figures 7I, J**).

Additionally, a previous study has demonstrated that RPS16, an MRPS6-interacting protein, plays a role in inhibiting viral replication and increasing the expression of type I interferon when its expression is knocked down (39). SNF8 has been demonstrated to interact with IRF3 and CBP to enhance the activation of the interferon antiviral response. This suggests that SNF8 is necessary for the optimal induction of the IRF3-dependent innate antiviral defense mechanism (40). Previous studies have postulated that HCV inhibits the translation of ISG proteins at the ribosome and confines viral replication to cellular compartments that are not susceptible to antiviral IFN-stimulated effector systems (41).

**Figure 8:**
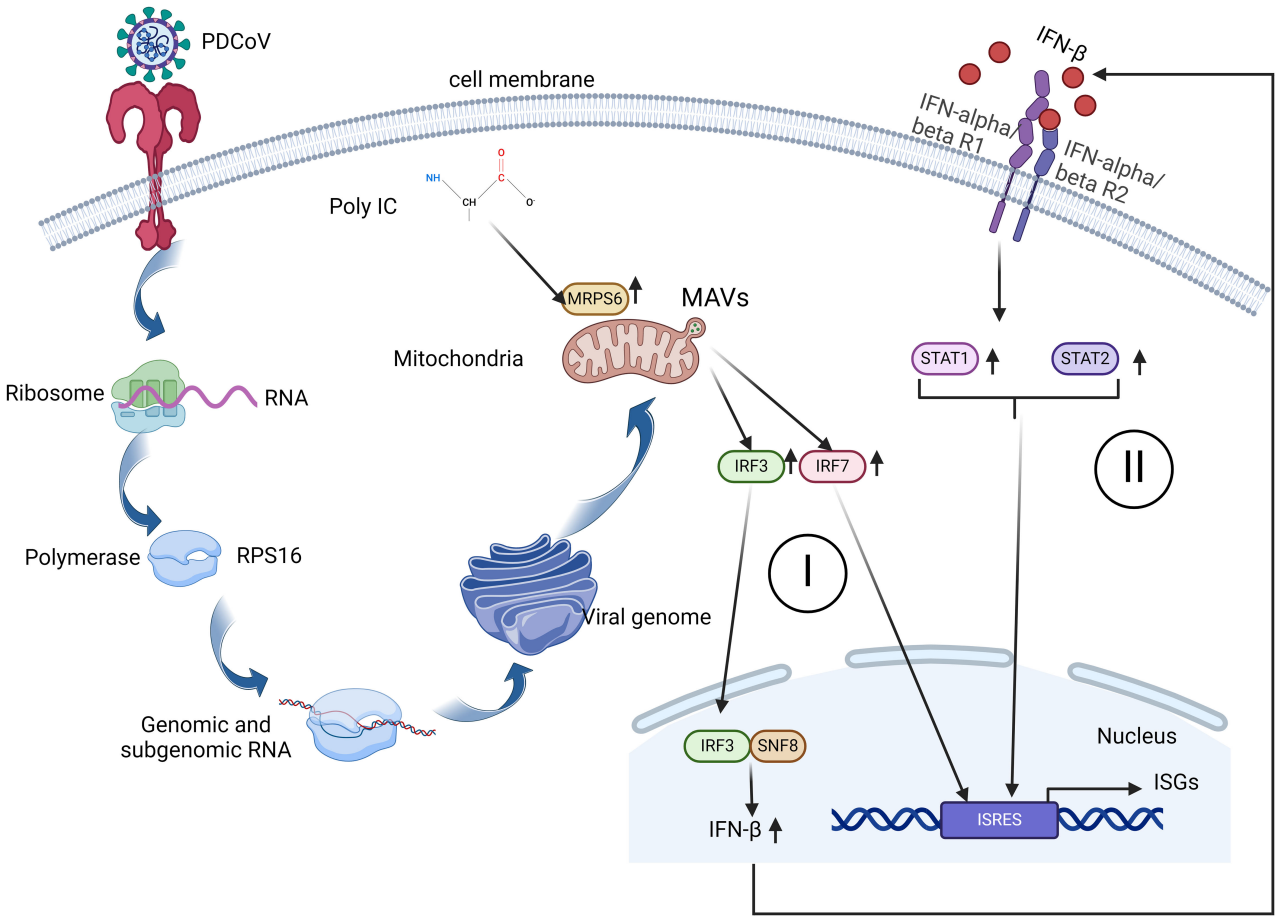
A schematic diagram of MRPS6 involvement in the PDCoV infection and interferon β pathways in the HIEC-6 cell line. Both PDCoV infection and poly-IC induce MRPS6 expression. Upregulated MRPS6 synergistically with the nucleic SNF8 induces IRF3, IRF7, STAT1/2, and IFN β protein production which are the main components of the IFN pathway. The next generation of IFN β would give a second wave of stimuli to strengthen interferon responses and produce more interferon stimulated genes.

In this investigation, we believe that PDCoV infected HIEC-6 cells internalize the virus through receptor-mediated endocytosis. Viral RNA translocates to the Golgi apparatus in the presence of RPS16, a protein known to interact with MRPS6. From the Golgi, viral RNA is subsequently transported to the mitochondria, where MRPS6, a subunit of the mitochondrial ribosomal protein, facilitates the translation of viral proteins. Because poly-IC stimulation to HIEC-6 cell leads to increased MRPS6 expression and an associated upregulation of the interferon-β pathway, we confirmed that MRPS6 is involved in the interferon-β pathway. Overexpression of MRPS6 further amplifies IRF-3 and IRF-7 activation, which enhances IFN-β production. The increase in IFN-β levels consequently reactivates their receptors, culminating in the upregulation of STAT1 and STAT2. Thus, this is a dual activation of interferon pathway coupled with both IRFs and JAK-STAT pathway, which present an obvious anti-PDCoV mechanism shown as **Figure 8**.

## 4 Discussion

PDCoV is a novel enteric coronavirus that exhibits infectivity in multiple host species including pigs, cattle, chickens, and humans (22, 42–44). However, the mechanisms underlying PDCoV’s ability to infect humans and its potential for human-to-human transmission remain elusive, presenting a significant challenge for disease prevention agencies.

PDCoV-infected HIEC-6 cells were used in the experiment, and previous workers have demonstrated that PDCoV-infected HIEC-6 experimental (30). We conducted a comprehensive analysis of the proteomic variations induced by PDCoV infection in HIEC-6 cells at different time points. Since there have been no previous studies on the proteome of PDCoV infection in HIEC-6 cells, we deemed it essential to investigate these changes to facilitate future research in this area. Our analysis revealed that PDCoV has a broad host range, capable of infecting multiple species. Subsequently, we collected HIEC-6 cells infected with PDCoV at 48h, as well as corresponding control samples, and performed proteomic analysis. Consequently, we further investigated the differential protein MRPS6, which exhibited the highest level of differential expression at 48 hours post-infection, for subsequent studies.

MRPS6, also known as C21orf101, MRP-S6, RPMS6, S6mt, and bS6m, is a mitochondrial ribosomal protein. Existing studies primarily investigate the role of MRPS6 in tumorigenesis, particularly in breast cancer, where it has been shown to exert tumor-promoting effects (45). MRPS6 gene was detected to be highly expressed after breast cancer surgery (46). No documented evidence exists in the verifiable literature regarding the antiviral effects of MRPS6. However, in light of the devastating impact of pneumococcal pneumonia in 2020, researchers have put forth encouraging guidelines proposing the potential efficacy of ribosomal antiviral strategies as a more promising therapeutic approach (47). In this seminal investigation, we provide the inaugural evidence substantiating the inhibitory impact of MRPS6 overexpression in HIEC-6 cells on PDCoV viral infection. Drawing upon the protein interactions of MRPS6, specifically RPS16, and SNF8, we postulate that the upregulation of MRPS6, concomitant with the downregulation of RPS16 or the upregulation of SNF8, engenders a hindrance in PDCoV viral replication.

In a prior study, it was elucidated that Toll-like receptors (TLRs) undergo activation after PDCoV infection in ST cells. Moreover, it was subsequently determined that the expression of TLR2 is augmented following PDCoV infection (43), as the activation of the TLR2 pathway during SARS-CoV-2 infection (48).In this study, HIEC-6 cells were infected with PDCoV. TLR2 on the surface of HIEC-6 cells facilitated the endocytosis of PDCoV and subsequently transported the virus to the Golgi apparatus using the ribosomal RPS16 transporter. Additionally, previous research has shown that RPS20 can inhibit the replication of CSFV in cells by regulating the activity of Toll-like receptor 3 (TLR3) (49). RRL10 acts as a direct downstream component of antiviral signaling in the antiviral defense pathway mediated by the bicistronic virus nuclear shuttle protein (NSP) interacting kinase (NIK) in plants (50, 51). Mitochondrial ribosomes serve as the site for protein translation within mitochondria, and MRPS6 facilitates the generation of mitochondrial antiviral proteins, MAVS, by influencing the translation of PDCoV proteins. Earlier studies have demonstrated that both MITA/STING and MRP exert substantial inhibitory effects on HBV replication *in vitro*. Additionally, MITA overexpression activates the IRF3-IFN pathway, implying that MITA/STING and MRP contribute to the regulation of HBV through the modulation of innate and adaptive immune responses (52). The ATP-binding transporter proteins MRP4 and MRP5 play a significant role in modulating the cellular export of drugs and signaling molecules (53). Overexpression of multidrug resistance-associated protein (MRP) hampers the induction of interferon-beta (IFN-β) mediated by the mediator of IRF3 activation (MITA) through the disruption of MITA-TBK1 interactions and the regulation of transcription factor 3 via TBK1-IFN (54). The generation of MAVS is instigated by PDCoV infection, resulting in a decrease in peroxisome abundance. PDCoV infection induces a diminution in peroxisome abundance, which serves as the cellular locus for MAVS-mediated IRF1 activation and type III interferons (IFN-III) production (28). Furthermore, Supraphysiological concentrations (16 μM) of selenomethionine (Se-Met), which led to increased expression of MAVS protein and phosphorylation of IRF3 (55), exhibited significant antiviral effects on PDCoV replication in LLC-PK1 cells. PDCoV infection induces the activation of MAVS, which subsequently activates the downstream molecule IRF3. Additionally, SNF8 acts in synergy with IRF3 and CBP to initiate the antiviral response mediated by interferon (IFN) (40). IRF3 localization in the nucleus is enhanced, facilitating IRF3 interaction with SNF8, thereby promoting the activation of the type I interferon (IFN) pathway, PGAM5 forms complexes with MyD88 and TRAF3, leading to the activation of the IFN I signaling pathway and subsequently inhibiting the replication of the PDCoV N protein (56). The researchers showcased that the NS7a protein of the PDCoV impedes the production of interferon-beta (IFN-β) by disrupting the interaction between IKKϵ and TRAF3, as well as interfering with the binding of IKKϵ to IRF3 (57). In this investigation, we hypothesized that MRPS6 possesses the ability to impede the infection caused by PDCoV by exerting regulatory effects on the interferon type I (IFN-I) signaling pathway. This modulation occurs through the targeting of IRF3, a downstream molecule of MAVS.

## 5 Conclusion

In this study, we performed a comparative proteomic analysis of HIEC-6 cells infected with PDCoV at 48h to simulate potential zoonotic infections in humans. To the best of our knowledge, this is the first proteomic analysis conducted using PDCoV-infected human intestinal cells. We also focused on the differential protein MRPS6 and investigated its expression and association with PDCoV infection. We observed that MRPS6 exhibited a certain antiviral effect against PDCoV infection, and the degree of PDCoV infection was correlated with the dosage of transfected MRPS6. We additionally performed initial experimental validation to support the potential inhibitory effect of MRPS6 on PDCoV infection via the IFN type I signaling pathway. Furthermore, this experiment generated substantial proteomic data, providing a solid foundation for future research on potential zoonotic diseases.

## Data availability statement

The datasets presented in this study can be found in online repositories. The names of the repository/repositories and accession number(s) can be found below: PXD049713 (iProX).

## Ethics statement

Ethical approval was not required for the studies on humans in accordance with the local legislation and institutional requirements because only commercially available established cell lines were used. Ethical approval was not required for the studies on animals in accordance with the local legislation and institutional requirements because only commercially available established cell lines were used.

## Author contributions

YJ: Conceptualization, Data curation, Formal analysis, Software, Validation, Visualization, Writing – original draft. GZ: Conceptualization, Investigation, Methodology, Resources, Writing – original draft. LL: Methodology, Writing – original draft. JC: Formal analysis, Resources, Writing – original draft. PH: Investigation, Writing – original draft. ZG: Investigation, Writing – original draft. JH: Writing – original draft. ZX: Data curation, Writing – original draft. MW: Writing – review & editing. CL: Funding acquisition, Validation, Writing – review & editing. NJ: Conceptualization, Formal analysis, Funding acquisition, Methodology, Project administration, Resources, Writing – review & editing.

## References

[1] Ji W Peng Q Fang X Li Z Li Y Xu C. Structures of A deltacoronavirus spike protein bound to porcine and human receptors. Nat Commun. (2022) 13:1467. doi: 10.1038/s41467-022-29062-5 35304871 PMC8933513

[2] Hu B Guo H Zhou P Shi ZL. Characteristics of sars-cov-2 and covid-19. Nat Rev Microbiol. (2021) 19:141–54. doi: 10.1038/s41579-020-00459-7 PMC7537588 33024307

[3] Miller MR Braun E Ip HS Tyson GH. Domestic and wild animal samples and diagnostic testing for sars-cov-2. Vet Q. (2023) 43:1–11. doi: 10.1080/01652176.2023.2263864 PMC10614713 37779468

[4] Wang M Yan M Xu H Liang W Kan B Zheng B. Sars-cov infection in A restaurant from palm civet. Emerg Infect Dis. (2005) 11:1860–5. doi: 10.3201/eid1112.041293 PMC3367621 16485471

[5] Bosco-Lauth AM Hartwig AE Porter SM Gordy PW Nehring M Byas AD. Experimental infection of domestic dogs and cats with sars-cov-2: pathogenesis, transmission, and response to reexposure in cats. Proc Natl Acad Sci U.S.A. (2020) 117:26382–8. doi: 10.1073/pnas.2013102117 PMC7585007 32994343

[6] Richard M Kok A De Meulder D Bestebroer TM Lamers MM Okba NMA. Sars-cov-2 is transmitted via contact and via the air between ferrets. Nat Commun. (2020) 11:3496. doi: 10.1038/s41467-020-17367-2 32641684 PMC7343828

[7] Woolsey C Borisevich V Prasad AN Agans KN Deer DJ Dobias NS. Establishment of an african green monkey model for covid-19 and protection against re-infection. Nat Immunol. (2021) 22:86–98. doi: 10.1038/s41590-020-00835-8 33235385 PMC7790436

[8] Singh DK Singh B Ganatra SR Gazi M Cole J Thippeshappa R. Responses to acute infection with sars-cov-2 in the lungs of rhesus macaques, baboons and marmosets. Nat Microbiol. (2021) 6:73–86. doi: 10.1038/s41564-020-00841-4 33340034 PMC7890948

[9] Zumla A Hui DS Perlman S. Middle east respiratory syndrome. Lancet. (2015) 386:995–1007. doi: 10.1016/S0140-6736(15)60454-8 26049252 PMC4721578

[10] De Benedictis P Marciano S Scaravelli D Priori P Zecchin B Capua I. Alpha and lineage C betacov infections in italian bats. Virus Genes. (2014) 48:366–71. doi: 10.1007/s11262-013-1008-x PMC7089089 24242847

[11] Mohd HA Al-Tawfiq JA Memish ZA. Middle east respiratory syndrome coronavirus (Mers-cov) origin and animal reservoir. Virol J. (2016) 13:87. doi: 10.1186/s12985-016-0544-0 27255185 PMC4891877

[12] Zaki AM Van Boheemen S Bestebroer TM Osterhaus AD Fouchier RA. Isolation of A novel coronavirus from A man with pneumonia in Saudi Arabia. N Engl J Med. (2012) 367:1814–20. doi: 10.1056/NEJMoa1211721 23075143

[13] Woo PC Lau SK Lam CS Lau CC Tsang AK Lau JH. Discovery of seven novel mammalian and avian coronaviruses in the genus deltacoronavirus supports bat coronaviruses as the gene source of alphacoronavirus and betacoronavirus and avian coronaviruses as the gene source of gammacoronavirus and deltacoronavirus. J Virol. (2012) 86:3995–4008. doi: 10.1128/JVI.06540-11 22278237 PMC3302495

[14] Li G Chen Q Harmon KM Yoon KJ Schwartz KJ Hoogland MJ. Full-length genome sequence of porcine deltacoronavirus strain usa/ia/2014/8734. Genome Announc. (2014) 2(2):Me00278–14. doi: 10.1128/genomeA.00278-14 24723718 PMC3983307

[15] Lee S Lee C. Complete genome characterization of korean porcine deltacoronavirus strain kor/knu14–04/2014. Genome Announc. (2014) 2(6):e01191–14. doi: 10.1128/genomeA.01191-14 25428966 PMC4246158

[16] Janetanakit T Lumyai M Bunpapong N Boonyapisitsopa S Chaiyawong S Nonthabenjawan N. Porcine deltacoronavirus, Thailan. Emerg Infect Dis. (2016) 22:757–9. doi: 10.3201/eid2204.151852 PMC4806967 26982324

[17] Le VP Song S An BH Park GN Pham NT Le DQ. A novel strain of porcine deltacoronavirus in Vietnam. Arch Virol. (2018) 163:203–7. doi: 10.1007/s00705-017-3594-8 PMC7087264 29022111

[18] Duan C. An updated review of porcine deltacoronavirus in terms of prevalence, pathogenicity, pathogenesis and antiviral strategy. Front Vet Sci. (2021) 8:811187. doi: 10.3389/fvets.2021.811187 35097055 PMC8792470

[19] Chen Q Gauger P Stafne M Thomas J Arruda P Burrough E. Pathogenicity and pathogenesis of A United States porcine deltacoronavirus cell culture isolate in 5-day-old neonatal piglets. Virology. (2015) 482:51–9. doi: 10.1016/j.virol.2015.03.024 PMC7111688 25817405

[20] Ma Y Zhang Y Liang X Lou F Oglesbee M Krakowka S. Origin, evolution, and virulence of porcine deltacoronaviruses in the United States. Mbio. (2015) 6:E00064. doi: 10.1128/mBio.00064-15 25759498 PMC4453528

[21] Li Y Niu JW Zhou X Chu PP Zhang KL Gou HC. Development of A multiplex qrt-pcr assay for the detection of porcine epidemic diarrhea virus, porcine transmissible gastroenteritis virus and porcine deltacoronavirus. Front Vet Sci. (2023) 10:1158585. doi: 10.3389/fvets.2023.1158585 37008344 PMC10060962

[22] Jung K Hu H Saif LJ. Calves are susceptible to infection with the newly emerged porcine deltacoronavirus, but not with the swine enteric alphacoronavirus, porcine epidemic diarrhea virus. Arch Virol. (2017) 162:2357–62. doi: 10.1007/s00705-017-3351-z PMC7086908 28374120

[23] Li W Hulswit RJG Kenney SP Widjaja I Jung K Alhamo MA. Broad receptor engagement of an emerging global coronavirus may potentiate its diverse cross-species transmissibility. Proc Natl Acad Sci U.S.A. (2018) 115:E5135–43. doi: 10.1073/pnas.1802879115 PMC5984533 29760102

[24] Boley PA Alhamo MA Lossie G Yadav KK Vasquez-Lee M Saif LJ. Porcine deltacoronavirus infection and transmission in poultry, United States(1). Emerg Infect Dis. (2020) 26:255–65. doi: 10.3201/eid2602.190346 PMC6986833 31961296

[25] Zhuang Q Liu S Zhang X Jiang W Wang K Wang S. Surveillance and taxonomic analysis of the coronavirus dominant in pigeons in China. Transbound Emerg Dis. (2020) 67:1981–90. doi: 10.1111/tbed.13541 PMC7228218 32163661

[26] Zhang H Ding Q Yuan J Han F Wei Z Hu H. Susceptibility to mice and potential evolutionary characteristics of porcine deltacoronavirus. J Med Virol. (2022) 94:5723–38. doi: 10.1002/jmv.28048 35927214

[27] Yang YL Liu J Wang TY Chen M Wang G Yang YB. Aminopeptidase N is an entry co-factor triggering porcine deltacoronavirus entry via an endocytotic pathway. J Virol. (2021) 95:E0094421. doi: 10.1128/JVI.00944-21 34406863 PMC8513460

[28] Liu S Fang P Ke W Wang J Wang X Xiao S. Porcine deltacoronavirus (Pdcov) infection antagonizes interferon-Λ1 production. Vet Microbiol. (2020) 247:108785. doi: 10.1016/j.vetmic.2020.108785 32768229 PMC7331541

[29] Guezguez A Paré F Benoit YD Basora N Beaulieu JF. Modulation of stemness in A human normal intestinal epithelial crypt cell line by activation of the wnt signaling pathway. Exp Cell Res. (2014) 322:355–64. doi: 10.1016/j.yexcr.2014.02.009 24534551

[30] Cruz-Pulido D Boley PA Ouma WZ Alhamo MA Saif LJ Kenney SP. Comparative transcriptome profiling of human and pig intestinal epithelial cells after porcine deltacoronavirus infection. Viruses. (2021) 13(2):292. doi: 10.3390/v13020292 33668405 PMC7918119

[31] Li M Ramage H Cherry S. Deciphering flavivirus-host interactions using quantitative proteomics. Curr Opin Immunol. (2020) 66:90–7. doi: 10.1016/j.coi.2020.06.002 PMC7749055 32682290

[32] Shah PS Beesabathuni NS Fishburn AT Kenaston MW Minami SA Pham OH. Systems biology of virus-host protein interactions: from hypothesis generation to mechanisms of replication and pathogenesis. Annu Rev Virol. (2022) 9:397–415. doi: 10.1146/annurev-virology-100520-011851 35576593 PMC10150767

[33] Wang W Li J Fan B Zhang X Guo R Zhao Y. Development of A novel double antibody sandwich elisa for quantitative detection of porcine deltacoronavirus antigen. Viruses. (2021) 13(12):2403. doi: 10.3390/v13122403 34960672 PMC8703818

[34] Song L Chen J Hao P Jiang Y Xu W Li L. Differential transcriptomics analysis of ipec-J2 cells single or coinfected with porcine epidemic diarrhea virus and transmissible gastroenteritis virus. Front Immunol. (2022) 13:844657. doi: 10.3389/fimmu.2022.844657 35401515 PMC8989846

[35] Huang Da W Sherman BT Lempicki RA. Bioinformatics enrichment tools: paths toward the comprehensive functional analysis of large gene lists. Nucleic Acids Res. (2009) 37:1–13. doi: 10.1093/nar/gkn923 19033363 PMC2615629

[36] Szklarczyk D Gable AL Lyon D Junge A Wyder S Huerta-Cepas J. String V11: protein-protein association networks with increased coverage, supporting functional discovery in genome-wide experimental datasets. Nucleic Acids Res. (2019) 47:D607–13. doi: 10.1093/nar/gky1131 PMC6323986 30476243

[37] Pang Z Hao P Qu Q Li L Jiang Y Xiao S. Interferon-inducible transmembrane protein 3 (Ifitm3) restricts rotavirus infection. Viruses. (2022) 14(11):2407. doi: 10.3390/v14112407 36366505 PMC9696312

[38] Chen J Li P Zou W Jiang Y Li L Hao P. Identification of A novel interferon lambda splice variant in chickens. J Virol. (2023) 97:E0174322. doi: 10.1128/jvi.01743-22 36877044 PMC10062172

[39] Wu W Wang C Xia C Liu S Mei Q. Microrna let-7 suppresses influenza A virus infection by targeting rps16 and enhancing type I interferon response. Front Cell Infect Microbiol. (2022) 12:904775. doi: 10.3389/fcimb.2022.904775 35873150 PMC9301362

[40] Kumthip K Yang D Li NL Zhang Y Fan M Sethuraman A. Pivotal role for the escrt-ii complex subunit eap30/snf8 in irf3-dependent innate antiviral defense. PloS Pathog. (2017) 13:E1006713. doi: 10.1371/journal.ppat.1006713 29084253 PMC5679654

[41] Heim MH Thimme R. Innate and adaptive immune responses in hcv infections. J Hepatol. (2014) 61:S14–25. doi: 10.1016/j.jhep.2014.06.035 25443342

[42] Liang Q Zhang H Li B Ding Q Wang Y Gao W. Susceptibility of chickens to porcine deltacoronavirus infection. Viruses. (2019) 11(6):573. doi: 10.3390/v11060573 31234434 PMC6631122

[43] Jin XH Zhang YF Yuan YX Han L Zhang GP Hu H. Isolation, characterization and transcriptome analysis of porcine deltacoronavirus strain hnzk-02 from Henan Province, China. Mol Immunol. (2021) 134:86–99. doi: 10.1016/j.molimm.2021.03.006 33740580

[44] Lednicky JA Tagliamonte MS White SK Elbadry MA Alam MM Stephenson CJ. Independent infections of porcine deltacoronavirus among Haitian children. Nature. (2021) 600:133–7. doi: 10.1038/s41586-021-04111-z PMC8636265 34789872

[45] Oviya RP Gopal G Shirley SS Sridevi V Jayavelu S Rajkumar T. Mitochondrial ribosomal small subunit proteins (Mrps) mrps6 and mrps23 show dysregulation in breast cancer affecting tumorigenic cellular processes. Gene. (2021) 790:145697. doi: 10.1016/j.gene.2021.145697 33964376

[46] Lin X Guo L Lin X Wang Y Zhang G. Expression and prognosis analysis of mitochondrial ribosomal protein family in breast cancer. Sci Rep. (2022) 12:10658. doi: 10.1038/s41598-022-14724-7 35739158 PMC9226049

[47] Rofeal M El-Malek FA. Ribosomal proteins as A possible tool for blocking sars-cov 2 virus replication for A potential prospective treatment. Med Hypotheses. (2020) 143:109904. doi: 10.1016/j.mehy.2020.109904 32502901 PMC7834321

[48] Planès R Bert JB Tairi S Benmohamed L Bahraoui E. Sars-cov-2 envelope (E) protein binds and activates tlr2 pathway: a novel molecular target for covid-19 interventions. Viruses. (2022) 14(5):999. doi: 10.3390/v14050999 35632741 PMC9146335

[49] Lv H Dong W Qian G Wang J Li X Cao Z. Us10, A novel npro-interacting protein, inhibits classical swine fever virus replication. J Gen Virol. (2017) 98:1679–92. doi: 10.1099/jgv.0.000867 28721853

[50] Carvalho CM Santos AA Pires SR Rocha CS Saraiva DI MaChado JP. Regulated nuclear trafficking of rpl10a mediated by nik1 represents A defense strategy of plant cells against virus. PloS Pathog. (2008) 4:E1000247. doi: 10.1371/journal.ppat.1000247 19112492 PMC2597721

[51] Rocha CS Santos AA MaChado JP Fontes EP. The ribosomal protein L10/qm-like protein is A component of the nik-mediated antiviral signaling. Virology. (2008) 380:165–9. doi: 10.1016/j.virol.2008.08.005 18789471

[52] Liu S Zhao K Su X Lu L Zhao H Zhang X. Mita/sting and its alternative splicing isoform mrp restrict hepatitis B virus replication. PloS One. (2017) 12:E0169701. doi: 10.1371/journal.pone.0169701 28056087 PMC5215812

[53] Ritter CA Jedlitschky G Meyer Zu Schwabedissen H Grube M Köck K Kroemer HK. Cellular export of drugs and signaling molecules by the atp-binding cassette transporters mrp4 (Abcc4) and mrp5 (Abcc5). Drug Metab Rev. (2005) 37:253–78. doi: 10.1081/DMR-200047984 15747503

[54] Chen H Pei R Zhu W Zeng R Wang Y Wang Y. An alternative splicing isoform of mita antagonizes mita-mediated induction of type I ifns. J Immunol. (2014) 192:1162–70. doi: 10.4049/jimmunol.1300798 24391220

[55] Ren Z Jia G He H Ding T Yu Y Zuo Z. Antiviral effect of selenomethionine on porcine deltacoronavirus in pig kidney epithelial cells. Front Microbiol. (2022) 13:846747. doi: 10.3389/fmicb.2022.846747 35242124 PMC8886123

[56] Yang X Kong N Qin W Zhai X Song Y Tong W. Pgam5 degrades pdcov N protein and activates type I interferon to antagonize viral replication. J Virol. (2023) 97:E0147023. doi: 10.1128/jvi.01470-23 37882521 PMC10688367

[57] Fang P Fang L Xia S Ren J Zhang J Bai D. Porcine deltacoronavirus accessory protein ns7a antagonizes ifn-Β Production by competing with traf3 and irf3 for binding to ikkϵ. Front Cell Infect Microbiol. (2020) 10:257. doi: 10.3389/fcimb.2020.00257 32656094 PMC7326017

